# City Residents Play a Pivotal Role in Managing Global Food Security While Improving Human Health and Minimizing Environmental Footprints

**DOI:** 10.3390/nu16234176

**Published:** 2024-11-30

**Authors:** Jan-Olof Drangert

**Affiliations:** Department of Thematic Studies of Environmental Change, Linköping University, 581 83 Linköping, Sweden; janolof.drangert@gmail.com

**Keywords:** environmental footprint, food security, healthy diets, nutrient recycling, planetary boundaries, urbanization

## Abstract

Background/Objectives: Improved global data allow for a new understanding of what impact the food we produce, eat and dispose of has on the environment, human health and Nature’s resources. The overall goal is to guide decision-makers and individuals by providing in-depth knowledge about the effects of their dietary preferences on human and environmental health. Methods: The method is to investigate ways to reduce environmental degradation and to secure healthy food supplies in an urbanizing world, and to quantify the options. Results: Reviewed articles show that by eating less meat-based food and more plant-based and soilless food, as well as reducing food waste and recycling urban-disposed nutrients as fertilizers, we could reduce agriculture’s land requirement by 50% to 70% while still securing a healthy food supply. Less land under cultivation and pasture would reduce global emissions to air and water to a similar extent, and allow Nature to reclaim freed areas in order to catch more carbon and rejuvenate biodiversity. Thus, we could avoid further environmental degradation such as the current clearing of new fields needed under a business-as-usual regime. Presently, some 17 million people die each year due to poor diets, which is more than double the 7 million deaths since the onset of the COVID-19 pandemic. A return to more plant-based diets with unchanged intake of proteins but less calories, sugar, salt and fat combined with less red meat and ultra-processed food would reduce foremost non-communicable diseases by up to 20% and prolong life. The article suggests that the international focus has gradually turned to the food sector’s big contribution to climate change, biodiversity loss and harmful chemicals as well as to poor human health. It argues that this century’s rapid population growth and urbanization give urban residents a pivotal role in food’s impact on agricultural areas, which today cover half of the globe’s inhabitable areas. Their food demand, rather than the activities of farmers, fishermen or loggers, will guide remedial measures to be taken by individuals, industry and the public sector. A tool to calculate the potential environmental footprints of individual or societal measures is presented. Conclusions: Measures to make the agrifood sector more sustainable are still pending full recognition in international fora such as the UN COP Summits. Smart cities fitted with infrastructures to recycle macro- and micro-nutrients and organic matter have the potential to ameliorate human-induced impacts such as emissions to air and water bodies, crossing planetary boundaries, and polluting extraction of N (nitrogen), P (phosphorus) and K (potassium). Rapid results are within reach since dietary change and the turn-around time of nutrients in food is short compared to decades or centuries for recycled materials in cars or buildings.

## 1. Introduction

The international community is deeply engaged in finding remedies to greenhouse gas (GHG) emissions, biodiversity loss and the Earth’s limited resource base [1,2]—and to global health challenges [3]. The focus of this article is to investigate potential measures to reduce the sizeable food-related stress on both human health and the planet. Food-related activities contribute to global warming with erratic rainfall, water shortage, floods and food insecurity, and poor diets are estimated to cause an annual death toll of 17 million people [4].

The production of food requires energy, water and nutrients, and in conventional agriculture, these resources are drawn directly or indirectly from the sun, rainfall and soil—while some mineral nutrients are mined. Today, food production and consumption exert a dominant impact on six of the nine planetary resources boundaries: climate, land, freshwater, nutrients, biodiversity and man-made chemicals [2], and according to Gerten et al., we could only feed 3.4 billion people sustainably without transgressing planetary boundaries [5].

The food scientist Georg Borgström [6] pioneered the idea that meat and dairy products represent a wasteful use of resources and are also more harmful to human health than plant-based food. The argument lost power as the Green Revolution increased yields, but present-day global environmental challenges seem to revive the importance of diets. For instance, Aleksandrowicz and colleagues found a median reduction in GHG emissions, land and water use of 22%, 28% and 18%, respectively, across all fourteen studied sustainable diet types [7]. The vegan diet ranked as the best for both land use (−45%) and GHG emissions (−51%). Also, human health and life expectancy could be improved through dietary modifications [8].

Rapid population growth and unprecedented urbanization in this century from 3 to an expected 8.5 billion urban residents [9] mean that cities will dominate the demands for food and water from rural areas, and turn cities into hotspots of wastewater, sludge and other organic waste. With business as usual, the land area required to feed the world population in the year 2100 needs to be expanded by almost 80 percent, assuming no productivity gains. All other things equal, this would lead to serious consequences such as worsened global warming, shrinking rainforests and more beneficial insects being endangered [10,11], potentially made more damaging by negative feedback loops [2].

**Figure 1:**
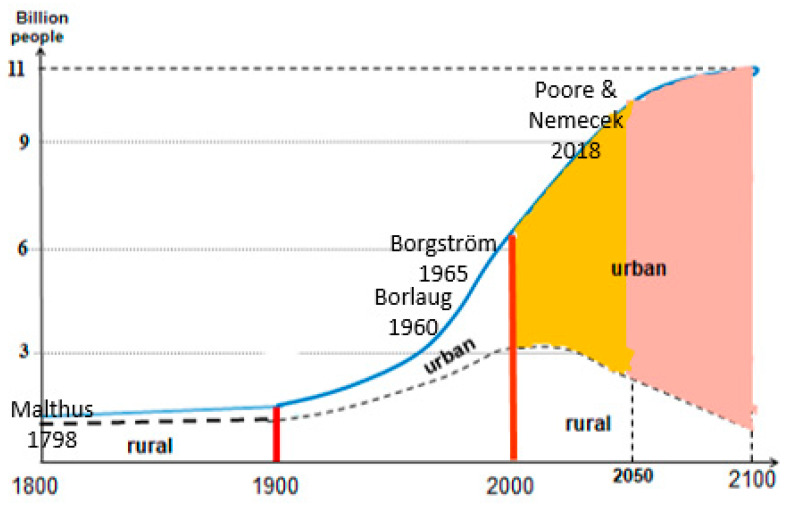
Development of the world’s actual and expected population from 1800 to 2100 in urban and rural areas. The yellow- and pink-shaded areas can be used as a proxy of the volume of excreta, urine or consumed food by urban residents in the first and second halves of the present century, respectively. The red vertical lines represent amounts of urine or food in the given years. Sources: Demographic data from [9,13].

The growth and distribution of the world’s population over three centuries, and its expected stabilization around 10–11 billion in this century are shown in Figure 1 [12]. The proportion of urban population was some 5% in the year 1800, and it is expected to reach 85% by the year 2100 [9]. Thus, the global concern is likely to focus on global environmental problems caused by substantial increases in consumption and waste disposal in cities, as well as on urban conservation, recovery and recycling of limited resources.

We may read Figure 1 as follows. Food production almost doubled during this century. A dot on the population curve can represent the volume of disposed urine in this year (e.g., red vertical lines), while the area between two years could represent the total food eaten in that period (e.g., colored areas). The graph tells, for example, that the volume of urine has trebled between the years 1900 and 2000, and that the amount of eaten food in urban areas is expected to increase by about 60% in the second half of the 21st century compared to the first half. The volume of disposed excreta during this century is estimated to be six times larger than in the 20th century, etc.

The graph may also depict the food-security discourse during various time periods. Malthus did not believe in yield increases and worried that the discrepancy between geometric population growth and an arithmetic increase in the agricultural area could only be eased by recurring famine and death [14]. George Borgström [6] pioneered more plant-based diets to reduce the need for more farmland. This was the time when Norman Borlaug’s work set the Green Revolution in motion with three- to four-fold increases in yields through new high-yielding, short-stemmed, rust-resistant wheat varieties combined with irrigation, pesticides and more fertilizers. The staple food winter wheat, for example, yielded only 2.7 t/ha in 1852–1871 and 3.07 t/ha in 1966/67 in Europe, but with the Green Revolution, yields increased to 5.48 t/ha in 1970–1975 and 8.69 t/ha in 1991/92 [15]. Today, cereal yields may still differ by a factor close to ten between wheat-producing countries [3].

Malthus’s fear in 1798 of famine was warranted from his vantage point (Figure 1) with low productivity in the early 19th century, and high mortality rates. The Green Revolution has been successful since the 1960s and has helped to provide enough but unequally distributed food produced on an almost unaltered total acreage [10]. A focus on food providing sufficient calories rather than protein in combination with increased consumption of fast-food diets has increased the non-communicable disease burden [16]. Today, worries are still with us but this time are mainly associated with obesity and diet-related diseases and with climate change, biodiversity losses and shortage of fertilizers [2,10]—each of which threatens to curtail agricultural output. Borgström’s idea to modify diets away from animal-based ones was set back by the Green Revolution, but now seems to gain ground.

The overall goal is to guide decision-makers and individuals by providing in-depth knowledge about the effects of their dietary preferences on human and environmental health in line with the UN Food and Agriculture Organization (FAO) definition of sustainable diets, which are healthy, have a low environmental impact, and are affordable and culturally acceptable. The objective is to investigate and quantify ways to reduce environmental degradation and to secure healthy food supplies in an urbanizing world.

## 2. Methods

Global perspectives drive the problem description and inform about what countermeasures there are, all of which are founded on a growing body of published ground-breaking studies, e.g., [5,7,8,16,17], and various UN body reports. Global data are used also because so much of the produced food and agricultural inputs are traded internationally by large corporations [18]. However, solutions to reduce our environmental footprints and to improve human health remain local or national.

Globally, human-induced degradation of the environment and unsustainable use of natural resources are recognized [5]. Climate change is an established fact [2], and six of the nine planetary boundaries identified by Rockström and colleagues [19] have now been crossed [2]. All six are closely connected to the food system and will be addressed here: climate change, land system change, freshwater change (green and blue), biogeochemical flows (N, P), biosphere integrity (biodiversity) and novel entities (chemicals). Yet, the food sector’s contribution is still pending full recognition in international fora such as the UN COP Summits.

Food is closely associated with human health and well-being. Today, nearly a billion people are undernourished while close to two billion are overweight or obese [20]. Obesity is rapidly increasing [10] but a new generation of diabetes and weight-loss drugs might be helpful and lead to a large and sustained reduction in body weight [21]. Too much sugar, salt and fat in diets contribute to several diseases [4]. A return to minimally processed, plant-based, fiber- and bioactive-rich foods would decrease so-called metabolism-related health risk factors such as high blood sugar and fat levels that underpin the surge in non-communicable diseases like heart disease, diabetes and cancer [22].

**Figure 2:**
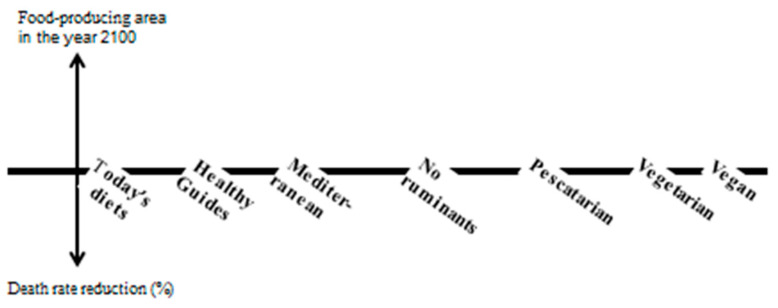
Conceptual framework connecting selected dietary options to human and environmental health.

Systems thinking is applied to address the nexus of food production, consumption (diets) and human and environmental health (e.g., biodiversity) [23,24]. Figure 2 shows the conceptual framework connecting selected dietary options to environmental and human health conditions. Human health is measured as death rates or added life years, while food-producing areas under agriculture and husbandry act as a proxy for GHG emissions, biodiversity and the use of fertilizers.

Today’s large variation in yields and GHG emissions among farms producing the same crop indicate that the agricultural sector could also potentially reduce negative environmental impacts [17]. Agriculture per se is not the focus of this article, despite its potential to contribute to food security and positive environmental impacts [25]. Nor is fishery in focus, despite serious overfishing [26], and the fact that farmed fish production overtook wild catch for the first time in 2022 [27].

Societal norms and individual perceptions influence the selection of diets [3] and daily routines among residents, and here viewed equally important as resource-saving technical and management arrangements. A major challenge is how to nudge urban consumers and guide food producers to jointly manage the agrifood systems to avoid unhealthy food, biodiversity loss, climate change and crossing of other planetary boundaries. An obvious time advantage for the food sector is that nutrients in the food have a turn-over period of weeks or months, and a person’s decision to modify diet or food waste management may even be shorter. Can cities with all their capabilities address such nexus problems in a sustainable way, while housing the extra 5.5 billion urban people in this century?

The chosen method is to identify sustainable patterns of food production and consumption as well as innovative urban infrastructure and house designs that could jointly protect and enhance human and environmental health. The goal is to provide decision-makers including individuals with practical tools to navigate toward a sustainable future.

## 3. Novel Findings About Food’s Role for the Global Environment and Human Health—And Potential Remedies

**Figure 3:**
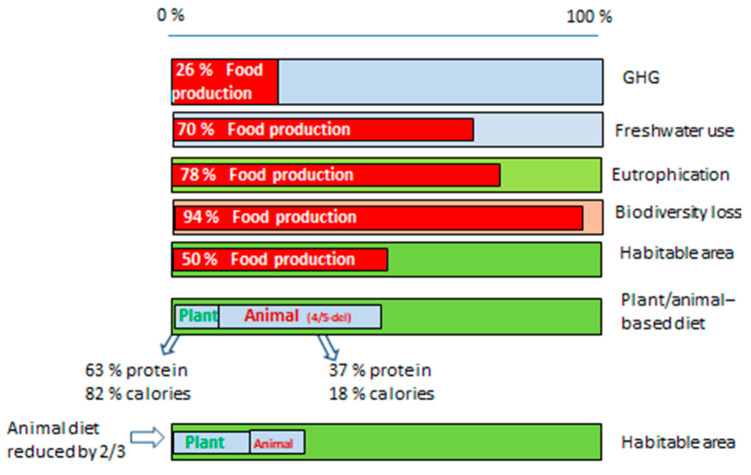
Food’s contribution (proportions in red) to global emissions and resource appropriation. An example of the effects of a 2/3 reduction in animal-based diets, while compensating with plant-based food to provide the same amounts of proteins and calories. Source: Created from data in [17].

Food production and consumption have become unsustainable in the process of feeding 8 billion people as evidenced by the agrifood systems contributing one-third of the total global GHG emissions [28] and transgression of global resources restrictions [5]. This article embraces a wide range of issues [23,24] that are relevant in order to remedy food’s negative impact on human and environmental health [10,16]. Our demand for certain diets is influenced by a variety of sociocultural and economic factors that in turn impact what the agricultural sector produces. Food impacts at least five fundamental environmental conditions (Figure 3): emits 26% of the total global GHG, occupies half of the globe’s habitable area, uses 70% of the world’s withdrawal of freshwater, contributes 78% of the globe’s eutrophication of freshwaters and oceans, and is responsible for 94% of the reduction in biodiversity biomass among mammals other than humans [17]. Thus, today’s food-related activities contribute to deteriorating human health and cause potential irreversible environmental damage.

In this Section, we first present various ways to improve health and reduce environmental damage caused by food-related activities and people’s willingness to implement such changes. The next Section highlights some adaptations of urban buildings and infrastructure that could support improved sustainability.

### 3.1. People-Centered Measures to Avail Healthy Food and to Protect the Environment

Every person contributes to shaping agrifood systems [29]. Residents can change their diets and ways to dispose of waste, while councils may enforce green building norms and new mobility systems, and industry and food distribution agents may provide more affordable and sustainable food products.

#### 3.1.1. Proteins and Calories (Energy) in Food

Since the Green Revolution, too strong an emphasis has been placed on calorie intake at the expense of the food’s protein content [16]. According to Poore and Nemecek, plant-based foods provide 63% of the consumed proteins and 82% of the calories, despite only occupying 23% of the global food-producing area (Figure 3) [17]. Such data challenge the common view that proteins are mainly found in meat and dairy products.

Soybean, for example, is a common ingredient in animal feed and plant-based food, and its protein content is about the same as of meat, some 15%, but beans need only one-tenth of the land area per produced kilogram of protein compared to meat [30]. Eating beans as an ingredient in a meal will therefore reduce land use and ameliorate environmental degradation proportionally compared to first feeding the beans to animals to be slaughtered and then prepared as food. Soybean-based products are already established on the market and do partly replace animal-based cow milk, cheese, butter and burgers.

#### 3.1.2. Diet’s Impact on Human Health

Non-sustainable diets include inadequate intake of fruit, vegetables, nuts and seeds and fibers together with high consumption of red meat and processed food. Such diets contribute to the burden of non-communicable diseases, and four out of the top five human health risk factors are diet-related [10].

Fadnes and colleagues generated dose–response relationships between mortality and intakes of 15 food groups in seven countries and estimated the life expectancy gains for 40-year-olds through a shift to optimized diets [8]. For example, following sustained changes from typical country-specific dietary patterns to longevity-optimized diets, the added years ranged from 6.2 years [5.7–7.5 years with 95% uncertainty interval (UI)] for Chinese females to 9.7 years [UI: 8.1–11.3] for United States males. Also, optimized vegan diets prolonged life by an extra 5.2 years [UI: 4.0–6.5] for Chinese females to 8.7 years [UI: 7.1–10.3] for United States males.

The Global Burden of Disease study of 195 countries estimated that 11 million deaths and 255 million DALYs were attributable to dietary risks in 2017: some 3 million deaths [1–5 with 95% uncertainty interval (UI)] and 70 million DALYs [UI: 34–118] due to high intake of sodium, 3 million deaths [UI: 2–4] and 82 million DALYs [UI: 59–109] due to low intake of whole grains, and 2 million deaths [UI: 1–4] and 65 million DALYs [UI: 41–92] due to low intake of fruits [31]. A poor diet contributes to abnormally elevated cholesterol or fats in the blood, stroke, kidney disease, certain cancers and fatty liver among other conditions [3]. The World Health Organization estimated that more than 39% of adults and 18% of children worldwide are obese or overweight, and obesity is directly linked to dietary patterns throughout the life cycle. Obesity, in turn, accounts for 44% of diabetes cases, 23% of ischemic heart disease cases, and 7% to 41% of various cancers [32].

According to Springmann and colleagues, a change in diet from meat to beans and pulses and more fruits and vegetables would improve human health [33]. They found that about half of global deaths could be avoided thanks to eating 56% less red meat and the other half thanks to a combination of eating 25% more fruit and vegetables while reducing consumption of calories by 15% (associated with decreases in the proportion of people being overweight and obese). These global averages comprise major regional differences.

An exclusive plant-based diet can potentially cause iron anemia, and deficiency in zinc, calcium and B_12_ because it contains much less bio-available minerals than meat due to hard-binding phytates [34]. However, mankind has rarely eaten, e.g., grains and beans “raw”, but prepared them through soaking, sprouting, fermenting, roasting, etc., whereby the essential nutrients/minerals are unlocked and made available for the body to absorb. Modern methods to soak grains in heated lactic acid can reduce up to 90% of the bindings [35].

#### 3.1.3. Moving Toward More Healthy Food

Many nations have published recommendations for healthy diets and sustainable food. The trend on the ground often seems to go in the opposite direction with increased consumption of unhealthy food, not least sugar, salt and fat [3,10]. The National Food Strategy in the UK, for example, found that 80% of processed food is cheap but unhealthy and, together with the growing calorie-rich junk-food diets causing obesity, represent big health risks [10]. Children too are increasingly exposed to cheap and readily available ultra-processed, energy-dense, nutrient-poor foods resulting in obesity [20].

Urbanization changes people’s relationship to food and urban residents buy food on offer in stores and/or food places. Factors such as status, affordability, easiness of preparation, etc., guide their choice of diets, while product promotion may prove more decisive than health aspects. The amount of salt, carbohydrates and fat is largely decided by the food industry and food chains rather than by households. Health authorities are increasingly worried about mounting public health expenditure due to unhealthy food, but this does not seem to translate into selecting healthier diets [36]. Ultra-processed food is on the rise, despite it not being addictive like sugar and tobacco. This suggests that authorities could be more active in both nudging residents to change diets and to support research and promote promising healthy food initiatives, e.g., by raising taxes on unhealthy and junk food [10].

#### 3.1.4. Lower Intake of Animal-Based Food Reduces Agricultural Land Use and Biodiversity Losses

Plant-based diets require less land area for agriculture than animal-based diets. For example, Aleksandrowicz and colleagues concluded, after reviewing 65 relevant studies among several thousand studies, that the relative difference in land use (m^2^/capita/year) between current average diets and sustainable dietary patterns was as follows: vegans reduced land use by 55% (6 studies), and vegetarians only slightly less, 51% (7 studies) [7]. More surprisingly, by replacing ruminants (releasing methane) with monogastric animals, the land required goes down by 37% (7 studies), while healthy diet guidelines only reduce land use by 20% (10 studies). The spread around these given median values is generally up to ±20%. Poore and Nemecek compared area requirements per year for the best tenth percentiles of different food products and found that the area required to produce 100 g of protein in meat from beef herd, dairy herd, pigs, and poultry and peas is 42; 7.9; 4.8; 3.8 and 1.2 m^2^ respectively [17]. A vegan, for example, replacing red meat with peas will reduce the cultivated area for 1 kg of protein from 320 to 12 m^2^.

Initial crude calculations with data in Figure 3, without considering differences in soil fertility or that grazing and growing fodder often takes place on marginal land, tell us that with a zero animal-based diet, the same amount of protein and calories can be produced on a 60% and 70% smaller total agricultural area, respectively. With a more realistic two-third cut of the animal-based diet, 42% and 48% smaller total areas, including the required extension of the area for plant-based food production, is enough to produce the same amounts of proteins and calories (bottom bar in Figure 3). This finding is in line with what, for instance, Helander et al. found for Germany—that dietary change could reduce cropland footprints by 43% for a Lancet reference diet and up to 48% for a vegetarian diet [37]. They also expressed the required cropland area in m^2^ per capita and year, being reduced from about 2200 m^2^ to about 1250 m^2^. Kesse-Guyot et al. present similar results for France [34].

Factors such as land use, pesticides and climate change affect biodiversity. Land use is here used as a proxy where more land under cultivation or for grazing corresponds to more biodiversity loss [7]. Assuming linearity as a first approximation of the magnitude of losses, a two-third reduction in animal-based diets would lower land under cultivation by 42–48% and more than 40% of biodiversity losses could be avoided.

#### 3.1.5. Effects of Reduced Animal-Based Diets on CO_2_ eq. Emissions

Aleksandrowicz and colleagues found that the relative differences in GHG emissions (kg CO_2_ eq./capita/year) between current average diets and sustainable dietary patterns were 45% lower for vegans, 33% lower when ruminants are replaced by monogastric animals and no dairy products, 32% lower for vegetarians, and only 10% lower for a Mediterranean diet [7]. Poore and Nemecek compared the best tenth percentiles of different food products and found that producing 100 g of protein in meat from beef herds, dairy herds, pigs, poultry and peas emits 20, 4.1, 4.6, 2.4 and 0.9 kg CO_2_ eq., respectively [17]. Red meat stands out, but so do peas by emitting only one-twentieth to one-third of any meat. Moreover, their data show that variations in inputs and impacts among both products and producers are high. Kesse-Guyot et al. also demonstrate similar benefits by shifting from beef and lamb to chicken meat [34]. They also calculated the resources input to produce 1 L of milk to correspond to 10 g of beef, while 8 L of milk is required to make 1 kg of cheese.

Such data point to the considerable potential to reduce foremost methane emissions while securing food supplies [38]. It has also sparked research to reduce the amount of methane with new animal-feed formulas. The benefit of reducing emissions from meat-based diets is even bigger when considering the opportunity to repurpose the freed land to enhance sequestering carbon by afforestation [10]. Further research is needed to document the actual and potential magnitudes of dietary change and its impact on emissions in different landscapes and cultures.

#### 3.1.6. Reducing Food Waste and Other Losses

Reduced food waste and losses is identified as a major possibility to reduce acreage and the damage to the environment, and to save on fertilizer use. A report by the Food and Agriculture Organization (FAO) estimated that around one-third of the food produced globally was lost or wasted; however, the authors acknowledged a lack of household food waste data outside of Europe and North America [39]. The UN Sustainable Development Goal 12.3 aims to halve food waste and reduce food loss by 2030. UNEP reports that out of the food waste generated in 2019, 61% came from households, 26% from food service, and 13% from retail [40]. Furthermore, levels of household food waste were found to be similar for high-income and upper- and lower-middle-income countries. Such excessive wastage of food can be reduced by upstream measures such as improved household practices (especially in the North), and also food handling, packaging, transport, storage, etc. [10].

Residents seem to have been rather unaware of the magnitude of food wasted while being in favor of not wasting [39]. This is changing now when authorities and environmental groups—and also some food companies—promote hands-on guidelines for consumers to reduce food waste [10]. Eating more of the produced food will reduce global land use and fertilizer input proportionally, and halving the waste would save up to 16% (half of one-third) of the land area and of fertilizers, assuming that plant and animal-based diets are affected proportionally. Meanwhile, reduced food waste also means that fewer nutrients are available for recycling.

#### 3.1.7. Soilless Food Production

The innovative use of algae and seaweed as ingredients in food and feed is promising. The protein content of algae is high, about 40%, and requires 87% less area than soybeans and 99% less water [41]. However, both algae and soybeans need a considerable nutrient input to deliver good yields. Another alternative method to make meat production less dependent on available land and water is to let earthworms or fly larvae process manure and organic waste into protein-rich animal feed [42]. Adult crickets and mealworm larvae need 5–10 times less feed than cattle to produce the same weight gain [43]. This resonates with the FAO’s aim to increase insect-based food production in order to feed the growing global population [44]. More research is needed to make algae, worms, insects and flies competitive on a large scale.

A more futuristic approach is to make, e.g., fat and oil molecules from CO_2_ in an energy-demanding chemical process [45]. Oil from palm-tree plantations could be replaced by industrially produced molecules that are identical to the ones produced by nature, and already used as an input for making margarine. Oil crops makeup 12% of global crop production [18] and could ideally be manufactured without soil and fertilizers [46]. Another development run by Nasa is to convert electric energy, air, and water into simple molecules that can be used as sources of energy to turn the carbon, nitrogen, hydrogen, and oxygen from air and water into more microbes that produce food molecules—including proteins, fats, carbohydrates and dietary fiber—in the form of safe, palatable foodstuffs with various flavors and textures [47].

There are successful plant-based and/or fermentation-derived alternative products: margarine replacing butter, soy-based drinks replacing cows milk and cultivated-meat and vegetable burgers replacing meat burgers. Soilless alternatives may be accepted by consumers since they resemble conventional products. The Food and Land Use Coalition anticipates that soilless food production alone could account for as much as 10% of the global protein market by 2030 and then scale up rapidly [48]. The think tank RethinkX predicts that by 2030, 50% of dairy and beef products will have been replaced by alternative proteins [10]. However, the present experience is that hundreds of pioneering companies producing alternative food products struggle with falling financial valuation and market expectations [49].

### 3.2. Community-Oriented Transformation of Management Arrangements and Infrastructure

Urbanization leads to geographical concentration of waste volumes of solid and liquid waste, thus facilitating collection and treatment. The sanitation sector has favored technical and management innovation to solve upcoming problems from more complex contents of waste flows. It took a century to begin keeping wastewater and stormwater separate in two pipes to reduce recurrent overflows at treatment plants. Today’s litigation of the big private water utilities in the UK for causing thousands of sewage spills annually proves that the problems are not yet solved [50]. Also, recycling or pre-treatment of industrial wastewater before being released to the municipal treatment plant is still in its infancy. In this Section, we present some alternative infrastructure and ways to productively manage material flows and to provide a framework for engaging urban residents in recycling [51,52]. The focus is on nutrient and water flows.

#### 3.2.1. Returning Urban-Mined Nutrients Back to Agriculture

The global economic reserves of some macro-nutrients contained in fertilizers are dwindling this century, in particular, mined potassium (K) and phosphorus (P), but also sulfur (S) and nitrogen (N) produced from air by using natural gas [19,53,54]. Also, agricultural soil is often deficient in organic matter, which reduces its water-holding capacity. To what extent could mining of urban-disposed organic matter offset demand for fertilizers? Almost all nutrients enter cities in the form of food and leave as nutrient-rich excreta and food waste. Such limitless urban-derived nutrients and organic matter from urban households, food service (including excreta) and retail represent an alternative nearby source of nutrients and organic matter. For example, the total amount of N, P and K in all toilet water in Sweden, before losses, amounts to 20, 35 and 46%, respectively, of the annually sold mineral fertilizers in that country [53]. Such high values are expected, since the human body essentially utilizes the energy in eaten food. The environmental benefits from using this source would be to reduce the production emissions otherwise caused by mining P and K [55,56] and the need for their long-distance transport, as well as to avoid eutrophication and biodiversity losses caused by N and P being released to nearby water bodies and oceans [2]. Adoption of this traditional source of nutrients and organic matter will require convincing regulatory agencies like the EU to be accepted.

There is also room for increased nutrient reuse efficiency in the agrifood system as indicated by an alternative way to present the link between human food intake and food production (Table 1). An adult Swede, for instance, needs to eat the equivalent of some 250 kg of cereals annually that require an agricultural NPK-input of some 7.5 kg. The table shows that the annual volume and composition of excreted urine and fecal matter could replace a substantial part of the NPK fertilizer input [57]. The volatile nitrogen in urine is the most important to contain.

#### 3.2.2. Harnessing the Chemical Society

A sanitation arrangement must be easy to use, but difficult to abuse. Modern piped systems, however, make material flows invisible and thereby easy to abuse [58]. Toxic or harmful fluids and solids in various products are not seen with the naked eye, and therefore residents may dispose of such items in the toilet, washbasin, sink or rubbish bin. Also, the municipal sewage is concealed in the ground, and after passing a treatment plant, the effluent is disposed of in a water body while the polluted sludge is likely to be landfilled or incinerated. A range of sanitation approaches other than just adding treatment steps are available and include a shift from what comes out of the sewer pipe to what is brought into the household initially and disposed of in the sewer, while accepting that the two are intrinsically intertwined [59].

The challenge posed by the ‘*emerging chemical society*’ has the innocent heading “novel entities” in the planetary boundary language. The entities include synthetic chemicals and substances such as micro-plastics, endocrine disruptors, insecticides, and nuclear waste. The use of these should be zero unless proven harmless, but many man-made substances have not been evaluated [2]. Well over 100,000 different chemical substances are currently in use in household products in Europe [60]. A number of substances have been banned over the years, but at least 900 potentially harmful substances remain to be evaluated in the EU [59]. All citizens face health risks from harmful manufactured chemical substances in products that they buy and dispose of [60,61].

In this century, the goal could be to create sustainable toxic-free food and nutrient chains [60]. Instead of solely trying to separate out the valuable nutrients from harmful or toxic wastewater or sludge, the new focus is expected to be to produce more bio-degradable consumer products that allow recycling of sludge and food waste nutrients back to agriculture [62]. But regulating a resourceful chemical industry has proved difficult [63], including chemicals, which, when combined, cause cocktail effects [59]. An alternative is to install a separate sewer from toilets in order to provide a comparatively clean nutrient-rich sludge that can easily be turned into a good fertilizer [64,65,66,67].

Now is a window of unprecedented opportunity to design a sustainable urban infrastructure since both homes and infrastructure for an anticipated additional 5.5 billion urban residents will be built during the 21st century. A dual infrastructure with a separate flow for disposed high-quality nutrients could be installed when building new towns, city districts and neighborhoods in this century. If so, two-thirds of the world population would have homes with sustainable nutrient management systems at the end of the century without heavy additional investments. The existing buildings in the year 2000, housing some 3 billion urban people, will anyway be refurbished during this century and could be fitted with an extra pipe for toilet water at a reasonable extra cost. A win–win situation is present, providing food security, recovered green areas and reduced harmful emissions to air, water and soil.

#### 3.2.3. Greening Urban Areas

Urban agriculture and green cities were common in many cities of the world. Smith and colleagues reported in 1996 that half of the food was produced within the city limits in, e.g., Dar es Salaam and Moscow [68]. Today, green plots are mainly found in suburban single-home gardens with ornamental plants and lawns. Thanks to positive human health outcomes, a budding renaissance of locally produced vegetables and fruits is noticed [3]. New geographic surveys such as Edmondson et al. found that there is more than enough urban land available within UK cities to meet the fruit and vegetable requirements of the population [69]. Stringer reported that 700,000 New Yorkers could obtain all their vegetable requirements produced on a total of 52,000 acres of available backyard space [70].

**Figure 4:**
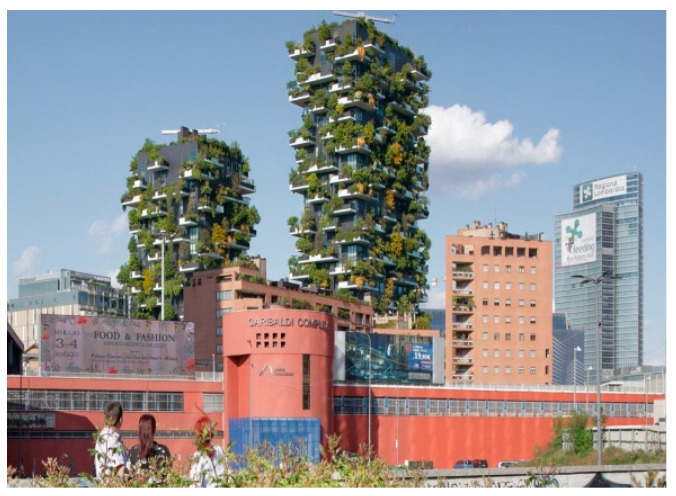
The El Bosco high-rise apartment buildings in Milan Italy with trees on balconies [71]. Photo: Paolo Rosselli (2017) by courtesy of Stefano Boeri Architetti.

**Figure 5:**
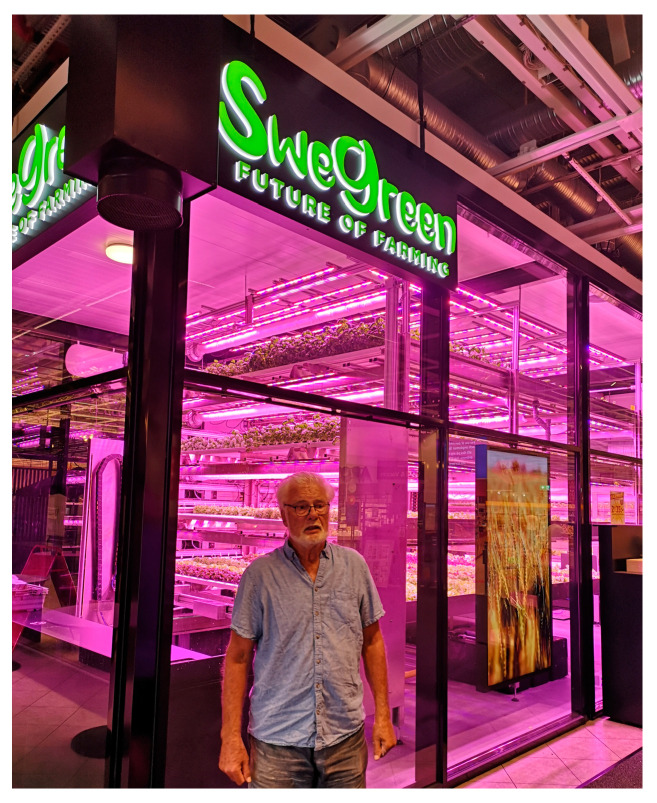
Greenhouse producing leafy vegetables inside a shopping center in Sweden. Photo: Jan-Olof Drangert.

Of late, a number of new apartments and public buildings have been fitted with roof gardens to retain rain and to produce vegetables. In Milan, Italy, two high-rise apartment buildings have balconies designed for bushes and small trees that reduce summer temperature and pollution (Figure 4). There are also advanced AI greenhouses inside shopping malls where customers can buy tomatoes, fresh salads and other leafy plants that are fed with nutritious water and electric light that replace the soil and sun (Figure 5). No pesticides are being used and 99% less water is required compared to conventional farming and no transport to the outlet. Other kinds of soilless food production are anticipated in urban factories in order to provide fresh food products.

In existing cities, the transport sector has had a profound impact through the expansion of private cars at the expense of other means of transport as well as green areas [72]. However, the favorable introduction of electric cars and bikes and improved public transport can be complemented with green food-producing areas that also make walking and biking more attractive. Broader priorities with new sustainability measures in city planning are taking prominence in order to improve human and environmental health, with the goal of sustainable water, sanitation and transit at the expense of space-demanding cars [73,74].

**Figure 6:**
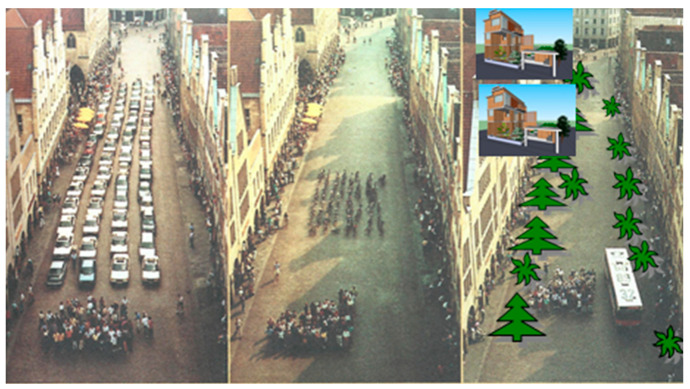
Roadway required by the same 80 passengers traveling by car, bicycle or bus. Freed city space allows for green streets and roofs. Source: Adapted from [76].

Figure 6 shows that space for greening purposes could also be made available in existing urban areas. Green flat roofs and walls may be used for food production and to catch and retard rain for conservation. As transit is being transformed, parts of streets and parking lots could be converted to green purposes. Work from home, digital meetings and other changes in lifestyle can further reduce the space needed for transport. In turn, this shift in urban land use will also save natural resources and reduce pollution, including air pollution by design [75].

## 4. Transformed Food Composition and City Infrastructure Will Change the Playing Field

### 4.1. Environmental Impacts

**Figure 7:**
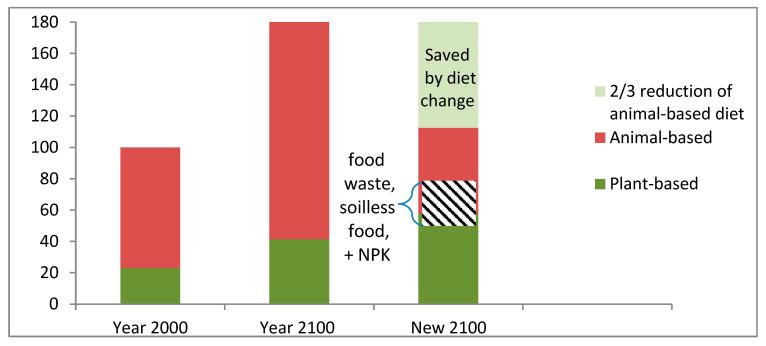
Required agricultural area to feed the world. Left bar: 23% plant-based and 77% animal-based food production in the year 2000. Middle bar: with business as usual in the year 2100, the required land area to feed 11 billion people is 80% larger than in the year 2000. Right bar: alternative with a 2/3 reduction in animal-based diets and an increase in plant-based diets while retaining an unaltered intake of protein and calories. Reduced food waste, soilless food products and increased use of urban-mined NPK can further reduce agricultural areas (striped area). Figure created with data from [17] and Section 3.1.4.

In the case of business-as-usual, vast up-to-now unexploited areas will be required to expand today’s agricultural production to feed 80% more people in this century, as shown by the middle bar in Figure 7. However, global dietary change has the potential to protect most of such areas from exploitation, as shown in Section 3.1.4. In addition, any combination of the other three measures (reduced food waste, soilless food production and recycled urban-derived nutrients) will allow a further substantial portion of already used agricultural areas to be reclaimed by Nature or forested areas. In this case, the required agricultural area in the year 2100 will be smaller than that in the year 2000. If afforested, the saved areas also become carbon sinks and give room for biodiversity gains.

A reduction in agricultural areas will simultaneously also contribute to a reduction in nutrient requirements, GHG emissions, biodiversity loss and eutrophication [7]. Table 2 summarizes the impacts of each of the four selected groups of measures on the three environmental factors of land and nutrient requirements and GHG emissions presented in the previous section. The conservative data represent magnitudes rather than exact numbers due to uncertainties and insufficient data. Local and national data are required in order to make locally informed policy and strategic decisions [5,77].

The impacts are interdependent in that any one measure will impact the outcome of the other measures [2,5]. At the present level of analysis, it suffices to avoid double counting. For instance, a one-third reduction in animal-based diets combined with halving food waste will result in a total saving of 32.8% = 20% + [100% − 20%] × 16% or = 16% + [100% − 16%] × 2 0% of agricultural land—and not 36% = 20% + 16%.

We assume a linear relationship between the impacts of each of the four measures in order to make predictions. The Formula (1) for the combined impact on land area change from a chosen combination of measures gives a rough estimate of the saved area—without double counting:

**Total potentially saved area** = X% of “40% ABD” + [100% − X% of “40% ABD”] × (Y% of “16% FWL”) + [100% − X% of “40% ABD” − [[100% − X% of “40% ABD”] × (Y% of “16% FWL)”]] × (Z% of “12% SLF”) + [100% − X% of “40% ABD” − [[100% − X% of “40% ABD”] × (Y% of “16% FWL”)]× (Z% of “12% SLF”)] × (W% of “10% RUN)” (1)

where X, Y, Z and W refer to the percentage change of each respective measure in Table 2 where “40% of ABD” = maximum area saved through a two-third reduction in animal-based diet; “16% of FWR” = maximum saved area through halving food waste; “12% of SLF” = maximum saved area through soilless food replacing 10% of meat and all vegetable oil; and “10% of RUN” = maximum saved area through increased fertilization with recycled urban-mined nutrients.

Information on potential outcomes of various measures will help policy—and decision-makers to predict the outcomes of selected actions and help decide recommendations accordingly. For instance, with X = 50, i.e., a one-third reduction in animal-based diets; Y = 100, i.e., halving food waste; Z = 50, i.e., replacing 5% of meat and half of oils with soilless products) and W = 20, e.g., recycling 20% of urban-mined nutrients, the formula tells that the total saved agricultural area is 37.2% (not 44% as by adding individual savings). Similar simple formulas, using data in Table 2, can be developed to calculate or predict combined outcomes from the four measures for GHG emissions and nutrient recycling.

### 4.2. Human Health Impacts

Several studies in the US and Europe show that the reduced intake of meat, in particular, red meat, favors good health: vegan diets fare best closely followed by vegetarian and pescatarian diets, and Mediterranean diets [7]. Fadnes et al. found a similar order pattern for extended longevity in seven countries [8]. Thus, health benefits similar to the above environmental gains can be reaped from increases in plant-based diets.

National and international bodies are burdened by increased costs for health systems due to increases in non-communicable diseases, and issue dietary recommendations and occasionally introduce legislation or taxes to guide what is produced and eaten [36]. Such initiatives are expected to impact people’s choices of diets. Red meat consumption is already in decline and replaced with chicken and pig meat [18], while health-threatening, highly processed and junk food is on the rise [10]. New drug-based treatments for obesity are beginning to influence obesity rates in the US and that may affect the urgency of dietary change for health reasons [21].

### 4.3. Joint Potential Impact

**Figure 8:**
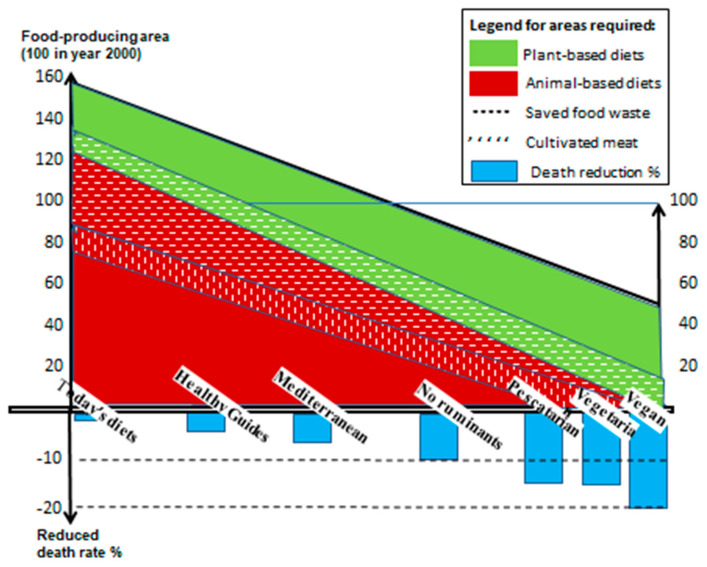
The impact of the three measures of dietary change, food waste reduction and soilless food production on the size of the agriculture area in the year 2100. Productivity increases achieved by recycled urban-mined nutrients as fertilizers would allow for even less land to be cultivated. Diet’s impact on death rates (% in blue). Sources: Inspired by [7,8].

Population growth in this century and business as usual will increase the required land area for food production from 100 to 160 units by the year 2100, of which 30 units are for plant-based and 130 units for animal-based diets. Figure 8 indicates that changing to more plant-based diets (green area) reduces total land requirements from 160 units with today’s diets to about 50 units if all people become vegans [17]. The required minimum area for each diet is the sum of the green and red (animal-based) areas in the figure. In addition, minimized food waste (--- areas) and a 10% increase in soilless food production (‘’’ areas) would reduce agricultural areas even further. Also, productivity increases in the sector by recycling urban-mined nutrients as fertilizers will allow for even less land to be cultivated, thus translating the lines further downward. In turn, such smaller agricultural areas will proportionally reduce GHG emissions and biodiversity losses.

Human health is also improved with more plant-based diets, as indicated by the average percentage reduction in rates of death in blue [7,8]. The figure demonstrates the pivotal impact individuals can have on their own health and their environmental footprints by changing diets and other measures.

## 5. Discussion

The next generation of urban planning and resource use is likely to be guided by a discussion focused on transforming agents, what residents can contribute, and the many actions that the public and business sectors can initiate from legislation to physical urban infrastructure in order to achieve potential future environmental and health gains.

### 5.1. Transforming Agents

Environmental degradation itself is a kind of transforming agent that forces the global society to take action. Climate change, deforestation, wildfires, shrinking ice caps and thawing tundra dominate the media. As for remedial actions, the energy and transport sectors come to the fore (e.g., [78]), while the worrying 34% of global GHG emissions caused by the agrifood system [28] receives less public attention. Therefore, this article highlights the great positive contribution the agrifood system can provide.

The UN General Secretary wrote in the invitation to the 2021 Food System Summit: ”It is time to change how we produce and consume, including to reduce greenhouse emissions.” Such statements are helpful in the processes of social change. Still, major changes also take place without any obvious guidance. One such transforming agent is the population increase that has contributed to the presently unsustainable agrifood system. Interestingly, though, urbanization itself has created (also unplanned) conditions forcing urban families to have radically fewer children than rural ones. Urbanization, thus, contributes to the stabilization of the number of people in the present century and will in turn provide some breeding room for revising what foods we produce and eat.

The ongoing social processes of female urban dwellers joining the out-of-home workforce and the proliferation of one-person households have pushed the demand for smaller dwellings and less time is spent on preparing food at home. These transforming agents promote a rapid expansion of, e.g., food services and the consumption of processed food—and the food industry’s influence on residents’ choice of diets [3].

The gender perspective also plays a role in societies’ selection of remedial environmental measures. The fact that energy and transportation are in focus may reflect that men are interested in developing new kinds of vehicles and energy systems—all of which belong to their traditional domain that provides engaging job opportunities. For instance, electric cars, hydrogen storage of energy, and even large-scale technical capture of carbon dioxide are in focus in the media, despite their limited impact on emissions compared to food interventions. Dietary issues, on the other hand, belong essentially to the female domain and are perhaps therefore likely to spark less attention. Presently, no attractive technology is available to change diets that would attract the media, perhaps with the exception of soilless food. Gender-oriented research could be strengthened to provide novel tools to promote food-oriented measures.

Another likely transforming agent is the limited nutrient resources for making fertilizers. The option to mine nutrients in urban food waste and excreta is polarized between those who would like to incinerate all sludge and organic waste, and extract P and K from the ashes (e.g., Singapore), and those who prefer to co-compost excreta and organic matter to produce biogas before manufacturing a fertilizer from the digestive. The first choice results in losing other valuable macro- and micro-nutrients as well as organic matter in exchange for some energy. The latter option requires a fairly clean sludge that can be made available by installing a separate sewer for black water from toilets or by securing that only toxic-free consumer products are disposed of by households [67]. This debate seems to be ending in the European Union with the new proposal to make it compulsory from 2024 to separately collect all bio-waste or recycle it at source, while existing incineration of sludge can continue [78].

Coming back to the transformative role that various UN bodies could play, the investigated food-related measures could ease some of the stalled issues at the UN COP 29 Summit. Two of their benefits are that dietary change and waste reduction are less costly and could be implemented more rapidly than many measures that are presently discussed in such fora.

### 5.2. Willingness to Adopt New Diets, Reduce Food Waste and Recycle Nutrients

Willingness to change differs over time and space, as do transforming agents, but is often attributed to improved economy, affordability, health issues and status [3]. Urban residents make individual or family choices of what food to eat while being nudged by culinary tradition, advertisement and perhaps also influenced by information about environmental and health impacts, etc. How willing are urban residents to change diets and other planetary boundary-impacting habits?

A first observation is that change is an ongoing process. Over the last two decades, for example, the world has experienced a 45% rise in meat consumption [18]. Since the world population has increased by 27% in the same period, the per capita increase in meat consumption is “only” 14%. More importantly, perhaps, almost the entire increase in meat consumption consists of the lower resource-demanding poultry and pork meat, while the per capita consumption of (unhealthy) red meat has gone down. Today, chicken meat (35%), pig meat (33%) and cattle meat (20%) dominate meat consumption [18] evolving from a situation where cattle meat dominated. Therefore, demand for more land and fertilizers per capita has increased only marginally and accompanying environmental benefits have been gained. A UK government report recently hinted that there are tentative signs that the world may be reaching peak meat consumption [10].

A shift back to more plant-based diets may be driven or delayed by taste, appearance, affordability and available processed food [3]. As for taste, food companies can for instance attract customers by adding (also unhealthy levels of) sugar, salt or fat to processed dishes. Health authorities may try to nudge citizens to prioritize certain foods in schools and other institutions and to use taxes to promote healthy foods. Food chains can promote sustainable customer choices and could play a crucial role in guiding buyers to healthy foods. However, companies may also fail to influence customers as the drop in ready-to-eat cereals shows, and health-conscious US consumers are swapping (sugary) breakfast cereals for smoothies, overnight oats and eggs [79].

More radical changes are seen in diets as becoming a vegetarian or vegan is on the rise in some developed countries. Also, some plant-based alternative ingredients are common on the international market such as margarine replacing butter, soy-based drinks replacing cows milk, and vegetable burgers replacing meat burgers. Soilless alternatives may also be accepted by consumers, since they resemble conventional products. Whether microbial-manufactured food could also be accepted is a more open issue, despite the fact that our bodies contain a few kilograms of indispensable microbes.

The United Nations with its 193 member states considers the provision of water and, from 2022, also basic sanitation as a human right. However, no clear responsibility is indicated, which gives rise to an unclear message as to whether authorities should provide such services, or whether residents become partners or solely responsible. How does this affect residents’ willingness to take some or no responsibility for resource-smart use of waste and to avoid polluting water while using various harmful products? An extra burden is placed on sanitation authorities to nudge residents to engage in recycling organic waste, and nudge or request city planners, architects and builders to construct easy-to-use sustainable infrastructure and residences. A successful case is the following recycling option with a joint responsibility. The city regulation taps well into present resident practice to sort organic solid waste for recycling. In Stockholm, for example, already 30% of the food waste is collected in order to produce biogas and make an organic product for soil improvement. The city’s waste company expects this rate to rise to 75% with the new EU rule [80]. However, will wastewater treatment companies become equally successful in recycling excreta, which is presently not legally allowed to be recycled in organic agriculture according to EU rules despite being recommended by the World Health Organization [64]?

### 5.3. Sustainable Buildings and Infrastructure

There is a variety of urban projects in northern Europe with separate blackwater collection and sludge treatment to fertilizer quality [65,67]. These show that urban buildings and infrastructure can be built to facilitate the recycling of urban-mined nutrients and prevent both the incineration of organic matter and the eutrophication of water bodies [81]. Recovered nutrients can enhance agricultural yields by partially replacing mined P and K and manufactured N and contribute all micro-nutrients and valuable organic matter in the organic waste. Such transformations, however, require new supportive legislation, sustainable sanitation arrangements and a reduction in the use of harmful chemical products [66].

A circular economy requires quality assurance of the flows. The European Chemical Agency (ECHA) requires that the manufacturer assess the environmental and health risks before introducing a new substance or product. This kind of self-control has had limited success [63]. An interesting new framework to reduce environmental degradation is the EU legislative proposal, Ecodesign for Sustainable Products Regulation [82], whereby all products should disclose detailed information in a “product passport”. This includes how the product is produced, what harmful chemicals it may contain, how the product can be repaired, recycled or disposed of, and so on. This will ideally give the consumer a chance to make informed choices and become a change agent. However, food and feed are excluded from these performance and information requirements, and this is likely to delay necessary agrifood changes due to inertia to promote the recycling of nutrients in excreta.

Some remedies to environmental and human health risks such as food waste reduction may make a difference instantly, while other remedies require prolonged implementation. For instance, during the 20th century, private cars gradually encroached on much of urban previously green areas and spared little space for land-based food production and recycling of nutrients. Reversing this development will take time and effort. A number of cities try other ways by encouraging urban horticulture on rooftops and vacant plots. One may also foresee the establishment of urban factories producing soilless food for the urban market. Cities in cooler climates may also envisage urban greenhouses producing fresh vegetables for the neighborhood the year around. Urbanization itself facilitates outreach with information on new diets and sustainable living, and the food chain could be guided by public and private sector initiatives [3]. Compulsory design changes in built-up areas and infrastructure and nudged changes in food composition would reduce land used for conventional food production and thus reduce biodiversity loss and other environmental degradation.

## 6. Conclusions

Climate change has made us realize that the food we eat affects the whole globe and our future living conditions. Yet, food only plays a marginal role in international fora such as the UN COP Summits on climate change or on harmful chemicals and biodiversity losses. Improved global data permit a new understanding of the impact of food that can guide individual behavior as well as industry and the public sector. Almost all people will live in urban areas, and food-related activities will directly impact conditions in half of the Earth’s habitable areas where the food is produced. The aim here is to analyze and quantify measures to reduce the current degradation of human and environmental health and provide individuals and decision-makers with relevant information to make informed food-related decisions for their future.

Eating less meat-based diets, reducing food waste and recycling urban-disposed nutrients as fertilizers could reduce agricultural acreage by 50% to 70% while still securing a healthy food supply. Thus, we could avoid the current clearing of land for new fields and pasture needed under a business-as-usual regime. At the end of this century, less land could be under cultivation than today and be reclaimed by Nature in order to catch carbon and rejuvenate biodiversity. Such measures will also ameliorate human-induced impacts such as emissions to air and water bodies, reverse crossing other planetary boundaries, and reduce polluting extraction of N, P and K. Rapid results are within reach since dietary change and the turn-around time of nutrients in food is very short: weeks rather than decades or centuries for recycled materials in cars or buildings.

A return to more plant-based diets with an unchanged intake of proteins and less calories, sugar, salt and fat combined with less red meat and ultra-processed food could reduce foremost non-communicable diseases and prolong life. Such changes in diets require joint efforts from dissemination of information, public participation and a cooperative food industry. Successful health measures will also enhance environmental health since most measures contribute to both human and environmental health.

Now is a window of unprecedented opportunity to design new sustainable urban infrastructure because an additional 5.5 billion urban residents will need both houses and infrastructure in the 21st century—and most have not yet been planned. If embarked upon now, two-thirds of the world population will have homes with sustainable nutrient management systems at the end of the century without additional investments. The existing urban buildings, housing some 3 billion people in the year 2000, will be refurbished during this century and could be fitted with an extra pipe for toilet water with little extra cost. A win–win avenue is wide open, providing food security, recovered green areas and reduced harmful emissions to air, water and soil.

## Figures and Tables

**Table 1:** Amounts of NPK-fertilizers needed to produce 250 kg of cereals, which corresponds to the required annual food intake by an adult, and the NPK content in (Swedish) feces and urine.

Important Macro-Nutrients	Urine 500 L/year	Feces 50 L/year	Total	Nutrient Need for 250 kg Cereal Yield
Nitrogen (N)	4.0 kg 88%	0.5 kg 12%	4.5 kg 100%	5.6 kg
Phosphorus (P)	0.4 kg 67%	0.2 kg 33%	0.6 kg 100%	0.7 kg
Potassium (K)	0.9 kg 71%	0.3 kg 29%	1.2 kg 100%	1.2 kg
Total amount				
of N + P + K	5.3 kg	1.0 kg	6.3 kg	7.5 kg

Source: [57].

**Table 2:** Global conservative estimates of reductions in agricultural land, nutrient requirements and GHG emissions achieved by four selected measures. Sources: [7,17,34] and Section 3.1 above.

Investigated Measure	Land Requirement (Max. Saved Area)	Saving of Nutrients (NPK-Fertilizers)	Global Reduction in GHG Emissions
2/3 reduction in animal- based diets (**ABD**)	37–40% (vegan diet needs 55% less area, and on 2/3 of 100%, i.e., in total 37%)	10% (estimated due to little synthetic NPK on grazing land)	8–10% (40% less cultivated land reduces global emission by 40% of 26%; alt. vegans emit 30% less of 26%)
Halving food waste and losses (**FWL**)	16% (half of 33% wastage)	16% (half of 33% wastage)	4% (half of 8% global emissions)
Soilless food replacing 10% of meat and half of all vegetable oils (**SLF**)	12% (−4% for oil crops, −8% for meat: 10% of 77%)	4% (−2% for oil crops and −2% for meat)	2% (−1% for oil crops and −1% for meat)
Recycling urban NPK- nutrients to fertilizer (**RUN**)	10% (thanks to estimated raised productivity)	30% (urban-mined NPK replacing synthetic)	1% (guesswork about reduced fertilizer production)

## Data Availability

The original contributions presented in this study are included in the article. Further inquiries can be directed to the author.
